# A first principles approach to differential expression in microarray data analysis

**DOI:** 10.1186/1471-2105-10-292

**Published:** 2009-09-16

**Authors:** Robert A Rubin

**Affiliations:** 1 Mathematics Department, Whittier College, 13406 E. Philadelphia St., Whittier, CA 90608, USA; 2 8620 Portafino Place, Whittier, CA 90603

## Abstract

**Background:**

The disparate results from the methods commonly used to determine differential expression in Affymetrix microarray experiments may well result from the wide variety of probe set and probe level models employed. Here we take the approach of making the fewest assumptions about the structure of the microarray data. Specifically, we only require that, under the null hypothesis that a gene is not differentially expressed for specified conditions, for any probe position in the gene's probe set: a) the probe amplitudes are independent and identically distributed over the conditions, and b) the distributions of the replicated probe amplitudes are amenable to classical analysis of variance (ANOVA). Log-amplitudes that have been standardized within-chip meet these conditions well enough for our approach, which is to perform ANOVA across conditions for each probe position, and then take the median of the resulting (1 - p) values as a gene-level measure of differential expression.

**Results:**

We applied the technique to the HGU-133A, HG-U95A, and "Golden Spike" spike-in data sets. The resulting receiver operating characteristic (ROC) curves compared favorably with other published results. This procedure is quite sensitive, so much so that it has revealed the presence of probe sets that might properly be called "unanticipated positives" rather than "false positives", because plots of these probe sets strongly suggest that they are differentially expressed.

**Conclusion:**

The median ANOVA (1-p) approach presented here is a very simple methodology that does not depend on any specific probe level or probe models, and does not require any pre-processing other than within-chip standardization of probe level log amplitudes. Its performance is comparable to other published methods on the standard spike-in data sets, and has revealed the presence of new categories of probe sets that might properly be referred to as "unanticipated positives" and "unanticipated negatives" that need to be taken into account when using spiked-in data sets at "truthed" test beds.

## Background

In this paper we introduce a very simple probe-level procedure for determining differential expression in single-color microarray experiments. It is not based upon any particular model for probes sets or gene expression, and depends on just two requirements for each probe set:

a) under the null hypothesis that a gene is not differentially expressed for specified conditions, for any probe position in the gene's probe set the probe amplitudes are independent and identically distributed (IID) over the conditions, and

b) at each probe position distributions of replicated probe amplitudes are amenable to classical analysis of variance (ANOVA).

After log transformation followed by within-chip standardization, the resulting within-chip standardized scores (z-scores) meet requirement a), and within chips, logs of probe values are reasonably well modeled as being gamma-distributed. Since ANOVA is quite robust with respect to the within-treatment distribution, b) holds as well. (Note that we could drop requirement b) and work with a nonparametric version of ANOVA. Classical parametric ANOVA is, however, more powerful than its nonparametric counterparts, so it makes sense to use it whenever feasible.) We hereafter assume that each CEL file's perfect match (pm) values have been log2 transformed, that a gamma distribution has been fit (using the CRAN R [1]*fitdistr *function) to the transformed data with the lower 0.1% and the upper 1% trimmed off, and that the log scores have been standardized by subtracting the mean of the gamma fit and dividing by the standard deviation of the fit. We will hereafter refer to the results of the transformation process as "standardized probe values" (or "within-chip standardized probe values" when it is important to make it clear that standardization does not take place across chips.) In order to focus on the "first principles" perspective and concepts presented here, we do not perform any background correction or normalization of probe sets in this paper. In practice, of course, doing such pre-processing prior to performing the ANOVAs could improve the effectiveness of the method when applied to experimental data. Within-chip standardization, however, has been carried out because it is in effect a general signal processing calibration procedure which ensures that probe amplitudes can be meaningfully compared across chips. It removes global chip effects which could otherwise be confounded with differential gene expression.

**Figure 1:**
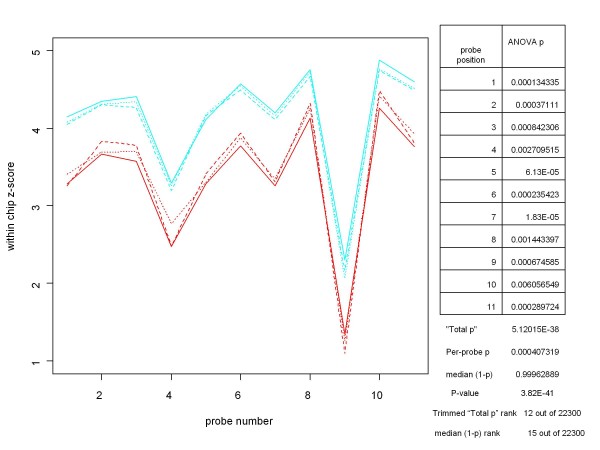
**An example of a standardized probe set plot, with probe-by-probe ANOVA results and summaries**. The left portion of this chart contains the probe set plot for the gene 205398_s_at from the HG-U133A Latin Square experiment for a large change in spike-in concentrations. Replicates are shown in cyan for 512 pM concentrations and in red for 256 pM. The table on the right lists the p-values for the ANOVAs performed at each probe position. "Total p" is the product of the probe-level ANOVA p's, and Per-probe p is the nth root of Total p, where n is the number of probes in the probe set. For this gene, rankings (among all genes on the six chips and two concentrations involved in the comparison) based the median of the (1-p)'s and on the trimmed Total p (i.e., the product of all but the highest and lowest of the probe-level p's) are comparable and consistent with the experimental design. The (unadjusted) P-value of the median was obtained directly from a beta distribution parameterized by the number of probes in a probe set.

Given a condition we wish to check for differential expression, we first limit the data set to be processed to those chips that are part of the condition. For each probe set we then proceed from one probe position to the next. At each probe position we perform analysis of variance (ANOVA) on the all of the standardized probe values at that position. We apply CRAN R's *aov *function and retain the p-value obtained from it. (The R *lm *function produces the same results, as would an independent two sample, equal variance t-test when two treatments are being analyzed for differential expression.) In Figure 1 we illustrate this concept with an example from the HGU-133A Latin square experiment [2]. In the left portion of the figure we display the standardized probe set replicates for the selected conditions, in this case gene 205398_s_at hybridized at 256 and 512 pM. The table on the right in the figure lists, for each probe position, the p-value resulting from performing analysis of variance on the probe values at that probe position. (Since we are comparing only two conditions here, we would have obtained the same p-value from independent two-sample, equal variance t-tests.)

Several ways of combining the probe level ANOVA p-values were examined. Perhaps the most conceptually appealing measure is the product of the p's. Under the null hypothesis that the gene under consideration is not differentially expressed for the specified conditions, it is reasonable to assume that the ANOVA results are independent from probe position to probe position. (Examination of the probe set plots for a small sample of non-spiked-in genes supports this assumption, as well as the assumption that probe amplitudes are IID across conditions under the null hypothesis.) In that case, the product of the p-values, referred to as "Total p" in Figure 1, is actually an over-all p-value for testing the null hypothesis. However, because there are a number of different probe set sizes on most arrays, it makes more sense to use the per-probe p-value instead; i.e. the geometric mean of the p-values (the nth root of the product of the p's, where n is the number of probes in the probe set). This allows for direct comparison of genes with different numbers of probes in their probe sets.

**Figure 2:**
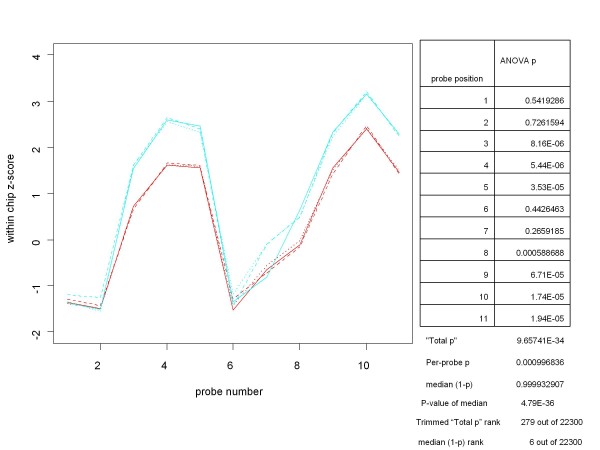
**An example of why the median of the (1-p)'s might be a better measure of differential expression than the (trimmed) Total-p**. This chart contains the probe set plot and ANOVA results for the gene 208010_s_at from the HGU-133A Latin Square experiment for concentrations of 128 pM (replicates in cyan) and 64 pM (replicates in red). The lack of a significant difference between conditions at probe positions 1,2, 6 and 7 adversely affects even the trimmed Total-p, while the median-based ranking of this gene (among all 22300 genes on the two chips involved in the comparison) is much more consistent with the Latin Square design. This is one among many cases for which the median seems to be the most robust measure.

In practice, however, as a tool for assessing differential expression Total p can be overly influenced by a few large (non-significant) probe level p-values, as can be the mean of the p's. Other summary measures such as the trimmed mean or trimmed geometric mean of the p's also do not appear to be as effective as the median in ranking genes in accordance with the known differences in concentration for the conditions being examined. As shown in the table in Figure 2, even after trimming the highest and lowest p-values, Total p can, in some cases, produce a much lower ranking of a condition than would have been expected. The Total p ranking of the comparison of 64 versus 128 pM concentrations for gene 208010_s_at from the HGU-133A Latin Square experiment was 279 out of the 22300 genes in the comparison. On the other hand, the rank based on the median of the ANOVA (1-p)'s for the same condition was 6, which is much more consistent with the concentrations involved. While determining the "best" way of combining the probe level p-values deserves a great deal more study, from this point on we will base our measure of differential expression on the median of the probe level p-values. In order to make a larger measure correspond to the condition of being more differentially expressed, our measure of differential expression for the gene will be the median of the probe level ANOVA (1-p)'s. This is a harmless change since the median of a set of (1-p)'s is the same as (1 - the median of the p's).

**Figure 3:**
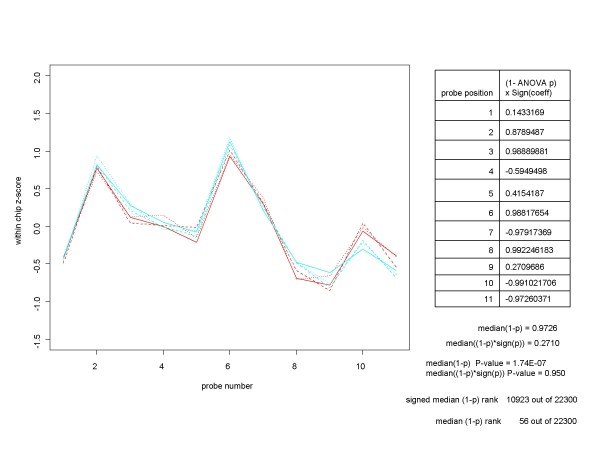
**An example of why the median of the signed (1-p)'s is a better measure of differential expression than the median of the unsigned (1-p)'s**. This chart depicts a common situation for non-spiked-in genes, such as 217207_s_at from the HGU-133A Latin Square experiment. The probe set plot and ANOVA results for the gene are shown for two of the experimental conditions, which we will designate by A (replicates in red) and B (replicates in cyan). At some probe positions the mean for condition A is larger than that for condition B, while the opposite is true at other probe positions. When we take this into account by multiplying each (1-p) by the sign of the ANOVA coefficient at that probe position before taking the median, we get a much better measure of differential expression for the gene.

Even though we can perform ANOVA on any subset (of size two or more) of the experimental conditions, in this paper we will restrict our attention to looking for differential expression between two conditions. For clarity, let us refer to the two conditions as A and B and suppose that we specify the ANOVA model such that a positive ANOVA coefficient, as provided by the *aov *function, corresponds to the mean response under condition B being larger (by the amount of the coefficient) than the mean under condition A. Under these conditions we consider a refinement of our procedure, in which each probe position's (1-p) is given the sign of the coefficient obtained from the across-replicates ANOVA for the probe. This additional step has two advantages. First, for many non-expressed genes, the ANOVA coefficient is positive at some probe positions and negative at others. For these genes, the median of the signed (1-p)'s will be closer in absolute value to 0 than will the median of the unsigned (1-p)'s. Thus, when we use the median of the signed (1-p)'s as the measure of differential expression, any gene with a score close to 0 is very likely not to be differentially expressed for the two conditions under consideration. Figure 3 provides an example of the improvement that can occur when signed (1-p)'s are used. For the experimental condition depicted, based on the median of the (1-p)'s the gene 217207_s_at, which is not spiked-in for the HGU-133A Latin Square experiment, ranks 56^th ^among the 22300 genes involved in this comparison. This is a higher ranking than that of 8 the 64 spiked-in genes. On the other hand, when we used the median of the signed (1-p)'s, the gene's rank is 10923, well below any of the spiked-in genes. Second, when a gene is differentially expressed between conditions A and B, the sign of resulting signed median tells us whether the gene is up-regulated or down-regulated. While that is not an important consideration in spike-in experiments, where the direction of the regulation is known beforehand, it can be very useful in real-world experiments.

In practice, when we work with signed (1-p)'s we take the absolute value of the median of the signed (1-p)'s as the measure of differential expression, retaining the median's sign in case we need to know whether the gene is up-regulated or down-regulated. The reason for working with the absolute value of the signed median is entirely pragmatic. If we retain the sign of median of the signed (1-p's) there could be two groups of differentially expressed genes - those with median near -1 and those with median near +1. There is no problem with that, but the two widely separated clusters of differentially expressed gene produce very non-standard looking ROC curves. In order to produce the familiar-looking ROC curves, we work with the absolute value of the median of the signed (1-p)'s. In this case, all differentially expressed genes have a score near +1.

The median ANOVA (1-p) approach described here readily lends itself to determination of an (unadjusted) p-value for the hypotheses test that a gene is not differentially expressed for the conditions under consideration. Under our assumptions for the non-differentially expressed condition, viz. a) at each probe position probe amplitudes are IID across the condition, and b) ANOVA results are independent from one probe position to another, it follows from a) that the ANOVA p-values at a single probe have a uniform distribution on the interval [0,1], and from a) and b) that the sample median of the ANOVA p-values over the probes in a probe set has a beta distribution whose two parameters depend on the number of probes in the probe set [3]. (More accurately, the median has a beta distribution only if there is an odd number of probes in the probe set. It is the mean of two beta distributions for probe sets with an even number of probes.) Regarding the absolute value of the signed median ANOVA (1-p)'s, under the null hypothesis the signed median has a uniform distribution over the interval [-1,1], so its absolute value also has a uniform distribution on [0, 1]. Thus its distribution under the null hypothesis is the same as that of the unsigned median.

**Figure 4:**
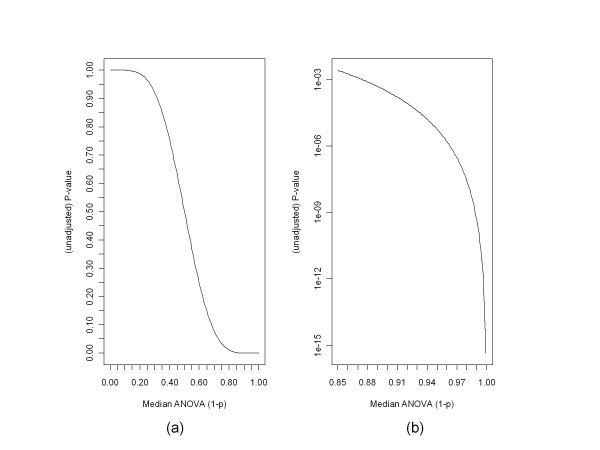
**P-values for testing the hypothesis of no differential expression as a function of the median ANOVA (1-p) scores for the case of 11 probes per probe set**. These charts show the unadjusted p-values corresponding to median ANOVA (1-p) scores for a specific number of probes per probe set. Similar curves can be obtained for any number of probes per probe set by means of the *pbeta *function in the R package *stats*, or the tools for working with sums of random variables in the R package *distr*. Figure 4(a) shows the full curve, while Figure 4(b) zooms in on the portion of the curve most involved with deciding on the question of differential expression.

Obtaining the p-value (unadjusted for multiple hypothesis tests) for a median ANOVA (1-p) score, x, is straightforward. For an odd number, n, of probes, p-value = 1 - *pbeta*(x, m, m) where *pbeta *is the beta cumulative distribution function in the CRAN R *stats *package and m = (n+1)/2. (For an even number, n, of probes, p-value = 1-p(x), where p is the cumulative distribution function of the mean of two beta distributions, (B(m,m+1) + B(m+1,m))/2, and m = n/2. We can obtain p(x) from the tools for working with distributions of sums of random variables found in the CRAN R *distr *package.) The unadjusted p-values for the median ANOVA (1-p) scores are shown in Figures 1, 2 and 3. Figure 4 shows the unadjusted p-values for the hypothesis test as a function of the median ANOVA (1-p) scores for the case of 11 probes per probe set, which is the case for the HGU-133A chip. Figure 4(a) shows the entire curve, and 4(b) shows the portion of the curve of greatest importance.

## Results

To assess the effectiveness of the ANOVA-p approach, we examined its performance on the three spike-in controlled experiments that are commonly used as test beds for differential expression procedures. These are the HGU-133A and HGU-95A Latin Square experiments [2] and the "Golden Spike" [4] experiment. For the two Latin Square designs we processed each contiguous pair of spike-in concentration conditions (referred to as the "d = 1" condition in McGee and Chen [5], and corresponding to a two-fold increase in concentration for most spiked-in genes). Since the Golden Spike experiment entails only two conditions - Control versus Spiked-in - there is only one comparison to consider.

### HGU-133A Latin Square experiment

As designed, the Affymetrix HGU-133A Latin Square experiment [2] selected 42 transcripts and assigned them to 14 groups with 3 transcripts each in each group. Each group was spiked-in at each of 14 concentrations from 0 to 512 pM, with 3 replicates per concentration. Within-chip and across-chip group concentrations were organized in a Latin Square design, i.e. within chips, concentrations increase by a factor of two from group to group (wrapping from 512 pM to 0), and for each group, concentrations similarly increase by a factor of two from one experimental condition to the next. See Appendix A of [5] for a complete description of the Latin Square design. The CEL files from this experiment, together with needed metadata, are available for download from the Affymetrix web site.

Researchers in the field have noted that several additional probe sets should be considered as spiked-in and have assigned those probe sets to groups with matching expression profiles [5]. We followed their recommendations and expanded the number known spiked-in probe sets to 64.

**Figure 5:**
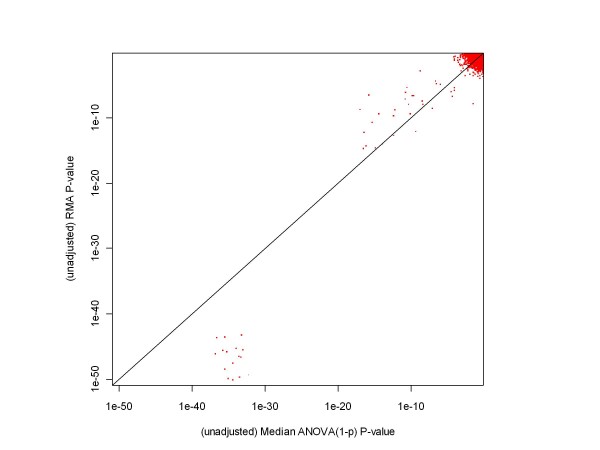
**A comparison of p-values obtained from median ANOVA (1-p) and RMA processing of a chosen pair of HGU-133A test conditions**. This chart shows that, based on their unadjusted p-values, median ANOVA (1-p) and RMA are in rather good agreement for unexpressed genes and genes with a factor of two difference in initial concentrations. There is quite a large difference in p-values for genes whose concentration changes from 512 to 0 pM, but that difference has no impact on whether the genes are declared differentially expressed or not.

**Figure 6:**
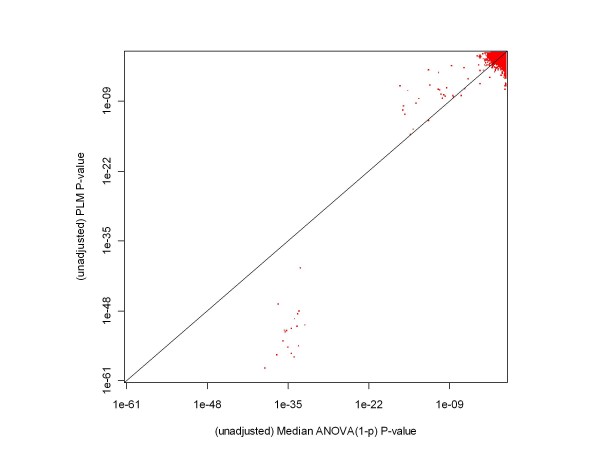
**A comparison of p-values obtained from median ANOVA (1-p) and PLM processing of a chosen pair of HGU-133A test conditions**. This chart shows that, based on their unadjusted p-values, median ANOVA (1-p) and probe level modeling (PLM) are in fair agreement for unexpressed genes and genes with a factor of two difference in initial concentrations. The tendency for median ANOVA (1-p) to produce, on average, somewhat smaller p-values for spiked-in genes and somewhat larger p-values for non-expressed genes may be due to inaccuracies in chip construction and/or probe level models. There is an even larger difference in p-values for genes whose concentration changes from 512 to 0 pM than for the RMA comparison, but again that has no impact on whether the genes are declared differentially expressed or not.

Figures 5 and 6 show the relationships between the unadjusted p-values obtained from our median ANOVA (1-p) methodology and those obtained from RMA and probe level modeling (PLM) processing of Experimental Conditions 1 and 2 of the HGU-133A Spike-in Experiment. (We chose those conditions as an example because, as mentioned above, there is a considerably expanded set of highly differentially expressed genes involved in the comparison.) RMA and PLM unadjusted p-values were obtained using the Bioconductor [6]*affylmGUI *package. As these plots indicate, the ANOVA-p approach produces larger p-values in the extremely low p-value region (the region associated with the genes whose concentrations changes from 512 pM to 0), but this difference has no meaningful impact - after adjustment for multiple hypothesis testing all of these genes remain highly significant regardless of the methodology applied. As for the other genes, ANOVA-p and RMA are in reasonably close agreement, and ANOVA-p and PLM are in fair agreement (median ANOVA (1-p)'s gives somewhat smaller p-values for spiked-in genes with two-fold concentration differences, and PLM has on average somewhat smaller p-values for the non-spiked-in genes). Since the receiver operating characteristic (ROC) curves shown in the following figures provide essentially the same information about the relationships between processing methodologies in a more easily interpretable format, we have not included any other scatterplots comparing p-values.

**Figure 7:**
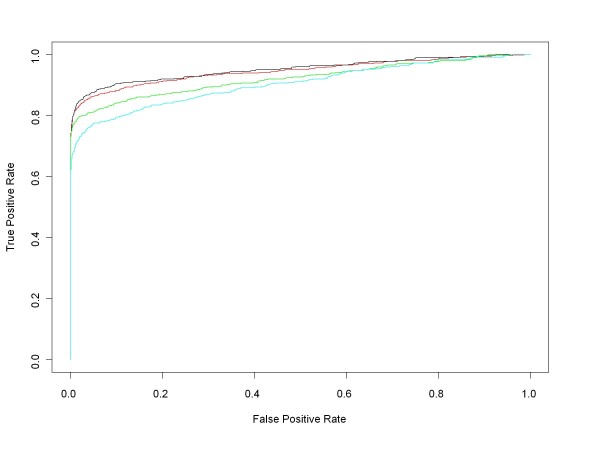
**Median ANOVA (1-p), Median Signed ANOVA (1-p), RMA, PLM ROC curves for all HG-U133A d = 1 conditions**. This chart shows the HG-U133A ROC curves for all comparisons in which experimental conditions increase by one (factor of 2) concentration step (plus those in the Latin square whose concentrations drop from 512 to 0 pM). ROC curves for each of the median of the probe level ANOVA (1-p)'s (red), the median of the probe level signed ANOVA (1-p)'s (black) and *affylmGUI's *RMA (green) and Probe Level Modeling (PLM) (cyan) were obtained after pooling the results of the analyses of all d = 1 conditions.

**Figure 8:**
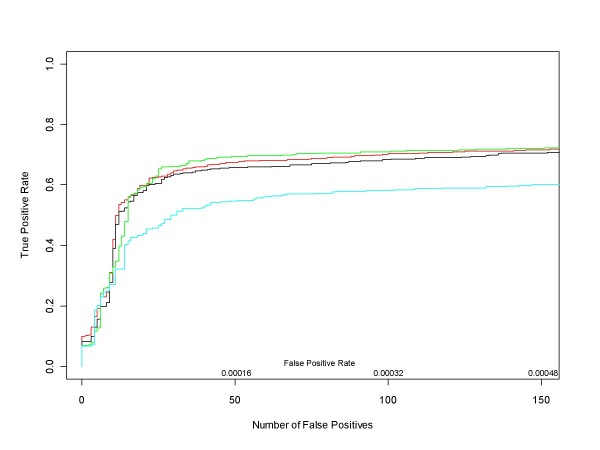
**Median ANOVA (1-p), Median Signed ANOVA (1-p), RMA, PLM ROC curves for all HG-U133A d = 1 conditions in the very low False Positive Rate region**. This is a close up view of Figure 7 in the region of highest real-world interest. Since the ROC curves were obtained after pooling the results of the analyses of all 14 d = 1 conditions in the Latin Square design, there are 311304 false positives in total. Median ANOVA (1-p)'s, median signed ANOVA (1-p)'s and *affylmGUI's *RMA and Probe Level Modeling (PLM) are shown in red, black, green and cyan respectively.

Figure 7 contains ROC curves comparing the performance of median ANOVA (1-p), median signed ANOVA (1-p), RMA and PLM over the full range of false positive rates for all d = 1 comparisons. These are comparisons in which experimental conditions increase by one (factor of 2) concentration step (plus those conditions in the Latin square where concentrations drop from 512 to 0 pM). For each d = 1 condition we obtained median ANOVA (1-p), median signed ANOVA (1-p), RMA and PLM scores for each of the 22300 HGU-133A genes. RMA and PLM scores were obtained using *affylmGUI *with default settings. Then, for each of the differential expression measures, we combined its 14 d = 1 results into a single data structure from which we calculated an ROC curve. Figure 8 shows the very short initial portion of the ROC curves up to 150 false positives (roughly an average of 10 false positives per d = 1 comparison), the region of highest practical importance.

### HGU-95A Latin Square experiment

The HGU-95A Latin Square design consists of 14 spiked-in human gene groups in 14 experimental groups [2]. The concentration of the 14 gene groups in the first experiment is 0, 0.25, 0.5, 1, 2, 4, 8, 16, 32, 64, 128, 256, 512, and 1024 pM. Each subsequent experiment rotates the spike-in concentrations by one group; i.e. experiment 2 begins with 0.25 pM and ends at 0 pM, on up to experiment 14, which begins with 1024 pM and ends with 512 pM. Each experiment contains at least 3 replicates. Replicates within each group result in a total of 59 CEL files. Most groups contain 1 gene, the exceptions being group 1, which contains 2 genes, and group 12, which is empty. (Specifically, transcript 407_at listed as present in group 12 is actually included in group 1.) See Table One of [7] for a tabular summary of the HG-U95A Latin Square design.

**Figure 9:**
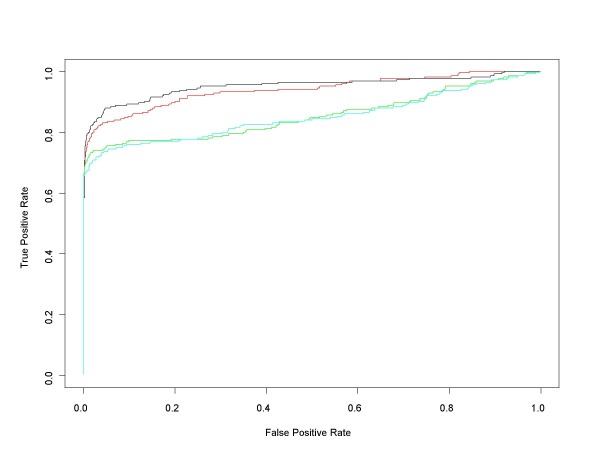
**Median ANOVA (1-p), Median Signed ANOVA (1-p), RMA, PLM ROC curves for all HG-U95A d = 1 conditions**. This chart shows the HG-U95A ROC curves for all comparisons in which experimental conditions increase by one (factor of 2) concentration step (plus those in the Latin square whose concentrations drop from 1024 to 0 pM). ROC curves for each of the median of the probe level ANOVA (1-p)'s (red), the median of the probe level signed ANOVA (1-p)'s (black) and *affylmGUI'*s RMA (green) and PLM (cyan) were obtained after pooling the results of the analyses of all d = 1 conditions.

**Figure 10:**
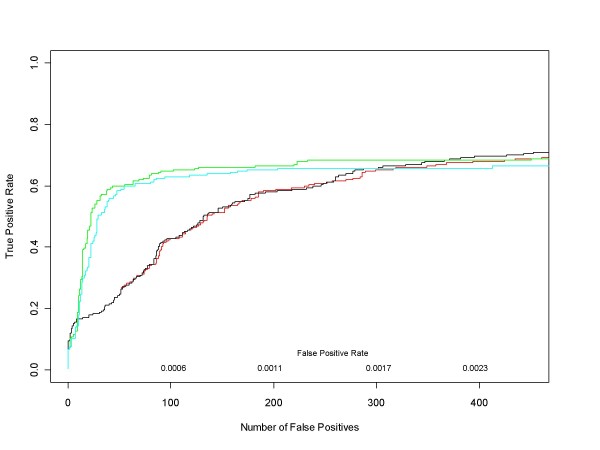
**Median ANOVA (1-p), Median Signed ANOVA (1-p), RMA, PLM ROC curves for all HG-U95A d = 1 conditions in the very low False Positive Rate region**. This is a close up view of Figure 9 in the region of highest real-world interest. Since the ROC curves were obtained after pooling the results of the analyses of all 14 d = 1 conditions in the Latin Square design, there are 176750 false positives in total. Median ANOVA (1-p)'s, median signed ANOVA (1-p)'s and *affylmGUI's *RMA and Probe Level Modeling (PLM) are shown in red, black, green and cyan respectively.

The ROC curves in Figure 9 compare the performance of median ANOVA (1-p), median signed ANOVA (1-p), RMA and PLM over the full range of false positive rates for all d = 1 conditions, obtained in the same manner as for the HG-U133A experiment. Figure 10 shows the initial portion of the ROC curves up to 450 false positives (roughly an average of 40 false positives per d = 1 comparison), the region of highest practical importance.

### "Golden Spike" experiment

The Golden Spike [4] experiment involved 3 Control and 3 Spiked-in Affymetrix DrosGenome1 GeneChip arrays, each of which has a total of 14010 probe sets. As described in [4], a total of 1331 probe sets had an increased concentration between the control and spike-in samples, 2535 probe sets had equal concentration and the remaining 10144 probe sets were empty on both the control and spike-in arrays. For the 1331 true positives, the log2 fold changes range from 0.26 to 2. The additional data files referenced in [4] provide access to a complete description of the experiment, as well as to the CEL files resulting from the experiment. Although the Golden Spike data set has been called into question by several authors [8,9], its value as tool for comparing differential expression methods is recognized even by its critics [8]. We thus included the Golden Spike data set in our explication of the ANOVA-p methods presented here.

**Figure 11:**
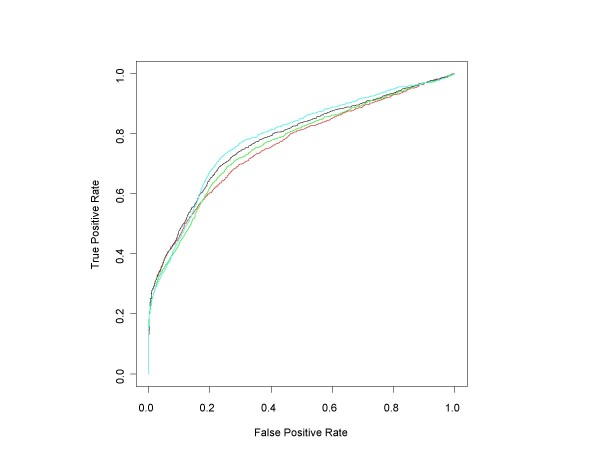
**Median ANOVA (1-p), Median Signed ANOVA (1-p), RMA, PLM ROC curves for the Golden Spike experiment**. This chart shows the Golden Spike ROC curves, where probe sets that were either "equal" or "empty" (in the terminology of Choe [4]) under both Control and Spiked-in conditions were considered to be false positives, while those with increased concentrations in the Spiked-in chips were taken to be true positives Median ANOVA (1-p)'s, median signed ANOVA (1-p)'s and *affylmGUI's *RMA and Probe Level Modeling (PLM) are shown in red, black, green and cyan respectively.

**Figure 12:**
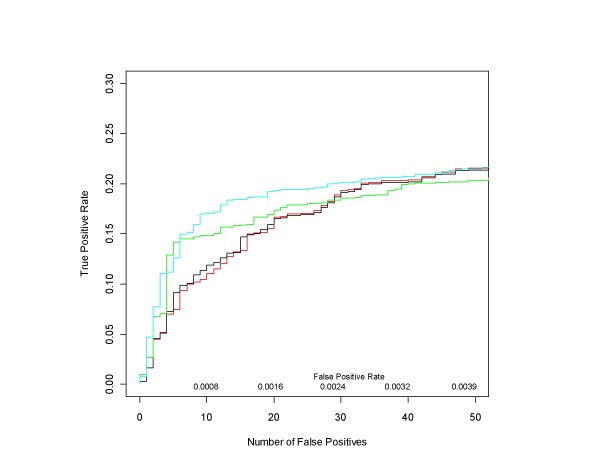
**Median ANOVA (1-p), Median Signed ANOVA (1-p), RMA, PLM ROC curves for the Golden Spike experiment in the very low False Positive Rate region**. This is a close up view of Figure 11 in the region of highest real-world interest. Since probe sets which were designated as either "equal" or "empty" were considered to be false positives, there are 12679 false positives in total. Median ANOVA (1-p)'s, median signed ANOVA (1-p)'s and *affylmGUI's *RMA and PLM are shown in red, black, green and cyan respectively.

The ROC curves in Figure 11 compare the performance of median ANOVA (1-p), median signed ANOVA (1-p), RMA and PLM over the full range of false positive rates for the Golden Spike experiment as described in [4]. Probe sets that were either equal or empty under both conditions were considered to be false positives, while those with increased concentrations in the spiked-in chips were taken to be true positives. Figure 12 shows the initial portion of the ROC curves up to 50 false positives, the region of highest practical importance.

## Discussion

### Spike-in Experiments, Differential Expression and Truth

In order to accurately assess the capabilities of procedures that decide which genes are differentially expressed, we need some completely characterized test data on which to base the assessments. There are special requirements for such "truthed" data sets, which are intended to be used to train or assess algorithms that will ultimately be applied to experimental data. The first and foremost requirement is that every probe set's condition and every comparison of a probe set across conditions in such a test bed be unambiguously assigned to some category, such as "Expressed" or "Not Expressed" ("Present" or "Absent" in Affymetrix MAS 5 terminology) in the case of a single condition, and "Differentially Expressed" or "Not Differentially Expressed" in the case of comparisons between conditions. Secondarily, measures of the degree of expression or differential expression for every condition, whether in the form of fold change or some other quantitative measure, are highly desirable. The various spike-in experiments were devised to provide that test data. Indeed, the spike-in experiments provide truth at the input stage of the experiment (i.e., accurate measures of the amounts of a variety of labeled probes in the mixes to be hybridized onto the chips).

The differential expression procedures discussed here and in a multitude of other publications do not however process the truthed hybridizing mixtures, which we will refer to as the "input truth" for the experiment. Instead these algorithms can only access the end result of the experiment, in the form of DAT or, more likely, CEL files. The process undergone by labeled probes from hybridization through scanning into DAT files to post-processing of the results to create CEL files is highly non-linear and not especially well characterized. Consequently "input truth" does necessarily translate into truth at the experiment's CEL or DAT file output stage, which we will refer to as the "output truth" of the experiment. Putting it more simply, knowing the difference between a probe set's initial concentrations across conditions does not guarantee that we know what the net effect on the resulting CEL files will be. There are many reasons why "input truth" might not translate to "output truth". Cross hybridization, SNP effects, incorrect sequencing of probe sets, probe set duplication and other types of erroneous probe set characterization have been identified as culprits. The use of BLAST and other on-line metadata and annotation sources has proved to be successful in making biologically-based corrections to the collection of probe sets that are expected to be differentially expressed in the CEL files. This situation has been recognized by several authors. For example, McGee and Chen [5] address the issue by adding in 22 new genes to the "truth set" for the HG-U133A spike-in experiment. Similarly, Affymetrix [2] points out the need to correct the list of HG-U95A spiked-in genes. Perhaps more critical to the results of spike-in experiments are the inaccuracies in the original probe set definitions that have been uncovered by more modern genomic techniques [10].

There is also a disturbing circularity with regard to truth and differential expression procedures in the literature. First, quite properly, analysis procedures are assessed based on their ability to get the "right answers" from the truthed data sets. On the other hand, what's considered "true" is in part based on the results of the data processing methods. For example in [5] McGee and Chen actually found 30 candidate new genes, but eliminated 8 of those for reasons that depend on which other genes were considered to be differentially expressed.

In this paper, in which we focus on first principles of statistical analysis and impose the fewest possible conditions on the data, we take a corresponding approach to the question of when a gene is differentially expressed. We consider a gene to be differentially expressed with respect to a specified test procedure if, for at least one pair of conditions in the experiment, we can reject the null hypothesis that the gene has the same distribution for the test statistic at a predetermined significance level, taking into account the multiple hypothesis testing environment in which the decision is being made. The adjusted p-value corresponding to the median ANOVA (1-p) score provides a quantitative indicator of whether the gene is expressed or not. Plots of the within-chip z-scores of the log amplitudes of the probes in a probe set provide an invaluable visual tool for judging whether the adjusted p-score, which is based up a summary measure of probe amplitude distributions, provides an accurate assessment of the behavior of the probe set. For example, consider the probe sets from the HG-U133A spike-in experiment with gene IDs 204890_s_at, 204891_s_at, 203173_s_at and 213060_s_at. These genes, along with four others, were addressed in McGee and Chen [5], but were not included in their new definition of the spiked-in data set. In the comparison of experimental condition 14 with experiment condition 1 (hereafter referred to as E14 vs. E1) of the HGU-133A Latin Square design, these four genes ranked 4, 5, 6 and 7 for differential expression, respectively, according to each of the unsigned and signed median ANOVA (1-p) approaches, RMA and PLM. The only genes that had higher scores were the three that belong to Group 1 -- the group for which there is a 512 to 0 pM change from condition 14 to condition 1. When we consider the combined results of all HGU-133A d = 1 comparisons (which involves a total of 896 spiked-in conditions), we find that these same four genes are among the top 100 genes for the ANOVA (1-p) approaches, RMA and PLM. Furthermore they are the only such putatively non-spiked-in genes in the top 200 results for any of the methods.

**Figure 13:**
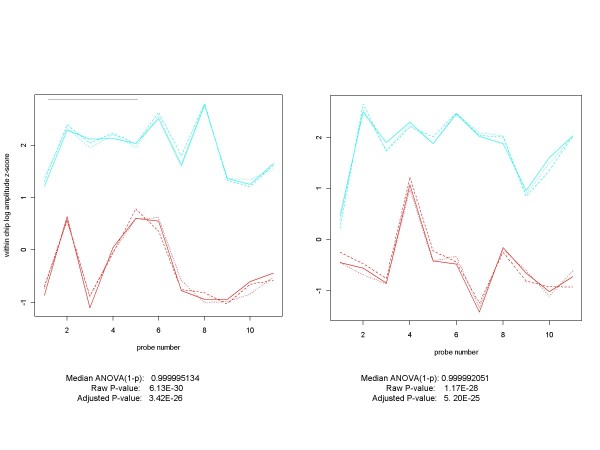
**Plots of within-chip log amplitude z-scores for two HG-U133A Spike-in Experiment "Unanticipated Positives"**. These two genes, 204890_s_at (shown on the left) and 204891_s_at (on the right) are neither in the original list of spiked-in genes for the Affymetrix HGU-133A Latin Square experiment, nor in the expanded list created by McGee and Chen [5] (although they considered including them but chose not to). However, as shown in this comparison of experimental conditions 14 (replicates in red) and 1 (replicates in cyan) of the Latin Square design, these genes are obvious candidates for a category we call "unanticipated positives". It is very difficult to image that these profiles could have come from probe sets with identical probe-level distributions for the experimental conditions 1 and 14.

**Figure 14:**
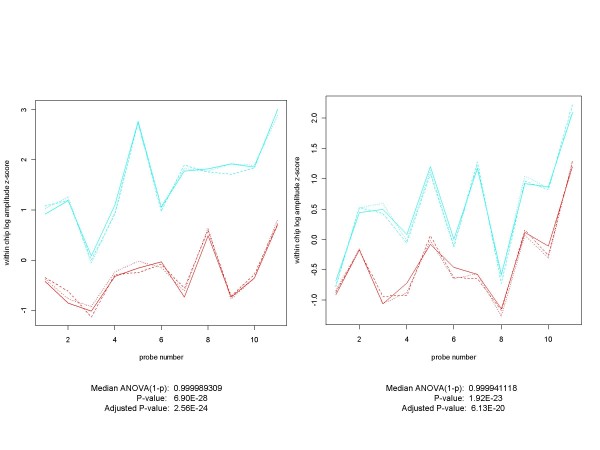
**Plots of within-chip log amplitude z-scores for two more HG-U133ASpike-in Experiment "Unanticipated Positives"**. These are two more genes which are not in the Affymetrix original list of spiked-in genes, and which were considered for inclusion by McGee and Chen but ultimately rejected. 203173_s_at (shown on the left) is similar in profile to 204890_s_at and 204991_s_at. The profiles are somewhat different for 203060_s_at (on the right), but still do not appear to be compatible with the hypothesis that they come from probe sets with identical probe-level distributions for experimental conditions 1 (cyan) and 14 (red). These genes are also obvious unanticipated positive candidates.

Figures 13 show the with-in chip z-scores for the probes in the 204890_s_at and 204891_s_at probe sets for the E14 vs. E1 condition. Figure 14 contains the E14 vs. E1 profiles for 203173_s_at and 213060_s_at. Although the separation between E1 and E14 conditions is not as great for 213060_s_at as it is for the other three genes, it is still extremely difficult to believe that these profiles arose from a gene with identical probe amplitudes distributions at each probe position for conditions 1 and 14 (and that all four quantitative measures examined in this paper are wrong in ranking these among the very highest of genes). We believe that from the perspective of an algorithm developer or assessor, these should not be considered false positives. Instead, since their differential expression was not predicted at the time the spike-in mixtures were prepared, we should call these "unanticipated positives", and not penalize any procedure that calls them differentially expressed. From an examination of these genes' profiles and quantitative scores over the various experimental conditions, it appears that they belong to Group 1 in the HGU-133A spike-in design.

**Figure 15:**
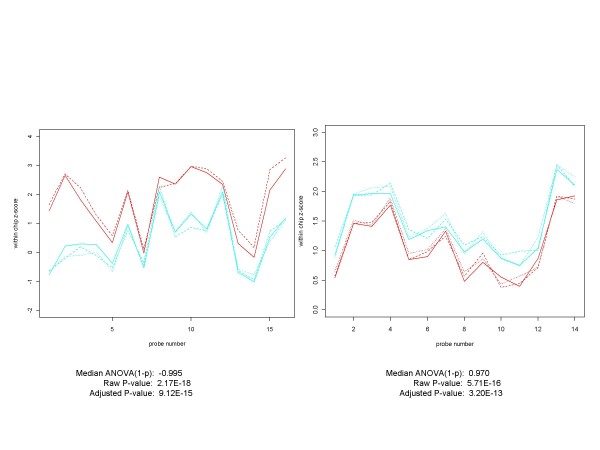
**Plots of Unanticipated Positives from HG-U95A and Golden Spike Experiments**. Unanticipated positives occur in all three spike-in experiments. Here are examples from each of the HG-U95A (on the left) and the Golden Spike (on the right) experiments. The non-spiked-in HG-U95A gene 32660_at ranks 2^nd ^out of 14010 genes (14 of which were spiked-in) in the experimental conditions C (replicates in red) vs. D (cyan) comparison for RMA, PLM and both of the median ANOVA (1-p) measures. For the Golden Spike plot, the non-spiked-in gene 142245_at ranked 23, 24, 177 and 66 according to median ANOVA (1-p), median signed ANOVA (1-p), RMA and PLM respectively, out of a total of 14010 probe sets, of which 1331 were designated as spiked-in. Plots for control arrays are in red, those for spiked-in arrays in cyan. In both cases the plots and p-values do not appear to be compatible with the hypothesis that they came from probe sets with identical probe-level distributions across conditions.

Unanticipated positives are found in all three of the spike-in data sets. Figure 15 contains examples from the HG-U95A and Golden Spike experiments.

The introduction of "unanticipated positives" underscores the conundrum we face with the use of these spike-in data sets as truthed test beds. On the one hand we would like to avoid subjective statements we have made, such as "it is still extremely difficult to believe that these profiles arose from a gene with identical probe amplitudes distributions at each probe position ...". In fact, if we had an unambiguously truthed data set to work with, we would not have to make any such statements. The problem is that there has been no established method for defining the truth in the very data sets that are used to assess the various algorithms applied to microarray data. If we use, say PLIER, to decide which genes "are" expressed or differentially expressed, then we should not be surprised if PLIER outperforms other methodologies on the resulting "truthed" data sets. Similarly using PLM, RMA, GCRMA, MBEI, etc to declare which are the truly expressed/differentially expressed genes, we should expect results to favor the procedure used to determine the "truth". Two ways of dealing with this problem come to mind. The first is to continue to use spike-in data sets in the current manner of use. They will still be very valuable tools in algorithm analysis, design and training, but they will never achieve their intended status as definitive test beds. The second is to make a community-wide effort to decide, even if somewhat arbitrarily in some cases, on the status of each comparative condition (or at least each d = 1 comparison) for each gene in the data sets. (It might be necessary to define an "Ambiguous" status to comparative conditions that do not achieve community-wide consensus, akin to Affymetrix "Marginal" status for probe sets on chips.) This might be an effort too large and/or contentious to undertake in practice, but something along those lines is needed if all algorithms are to have a level playing field on which to be compared.

**Figure 16:**
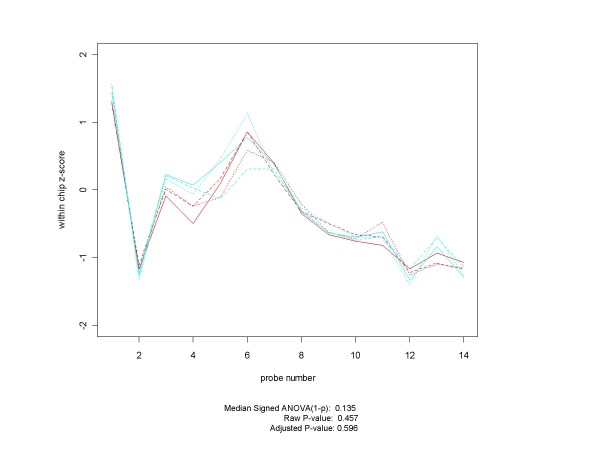
**Plot of a Golden Spike "Unanticipated Negative" with Fold Change = 3**. We also find some "unanticipated negatives" - genes which should be differentially expressed based on a large initial fold change, but which in practice do not appear to be. In the Golden Spike experiment 153553_at has a log 2 fold change of 3, yet it ranks 7246, 10007, 6034 and 6049 (out of 14010 genes in the comparison) using median ANOVA (1-p), median signed ANOVA (1-p), RMA and PLM measures, respectively. This plot of the within-chip z-scores (control arrays are in red, spiked-in arrays in cyan) and the corresponding p-values for the gene support the conclusion that 153553_at is a not differentially expressed and therefore an unanticipated negative.

**Figure 17:**
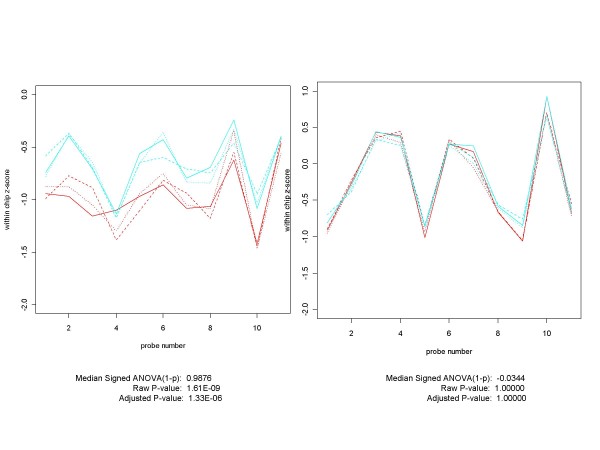
**Plots of comparisons involving lowest concentrations -- One differentially expressed, the other not**. These two genes, AFFX-r2-TagE_at (on the left) and 205790_at (on the right) are each spiked in at the lowest concentration (0.125 pM) in the Affymetrix HGU-133A Latin Square experiment. When this condition (in cyan) is compared with the non-spiked-in condition (in red), AFFX-r2-TagE appears to be differentially expressed while 205790_at does not. Using genes at low concentrations for assessing the effectiveness of differential expression algorithms presents some unique challenges. Sometimes, as for AFFX-r2-TagE, the gene is differentially expressed in the CEL files, in which case it is a suitable candidate for evaluating data analysis procedures. Frequently, however, as illustrated by 205790_at, the effect of the small initial difference in concentrations is wiped out by the many processing steps that occur on the way to creating CEL files. What results is a gene that is not differentially expressed at the CEL file level. It may be no easy task to identify which genes fall into which category.

When it comes to dealing with what would be considered false negatives from the perspective of the spike-in concentrations, the situation is a bit more complicated. First, there are some genes with high spike-in concentrations that should have manifested differential expression in the CEL files, but for some reason did not. For example, 153553_at is a gene in the Golden Spike experiment with a log 2 fold change of 3. Yet it ranks 7246, 10007, 6034, and 6049 (out of 14010) according to the median ANOVA (1-p), signed median ANOVA (1-p), RMA and PLM measures of differential expression, respectively. Its profile in Figure 16 and its p-values are that of a non-expressed gene. In this case we have what might be called an "unanticipated negative". Second, very low concentrations might have been included in the spike-in experimental design in order to assess the lower limits of sensitivity of the various differential expression algorithms, but they also serve to establish the lower limits at which differential expression actually occurs. Those genes for which differential expression does occur at the lowest concentrations do indeed provide the test bed for the sensitivity floor for any procedure. However, for many genes the numerous steps that take place after the preparation of the hybridizing mixture result in the gene not being characterized as differentially expressed in the CEL files. Because they are not actually expressed in the CEL files, these are not really "false negatives". Because it is no surprise that genes with very low concentrations wind up being non-expressed, they really aren't "unexpected negatives" either. For such genes one should not penalize an algorithm for not being able to distinguish a difference that existed at the start of the experiment but did not make it through to the final CEL file product. Figure 17 provides an example of each of these types of genes. The problem is how to tell one type of gene from the other in an efficient manner. Notice that in these cases, for which the probe set profiles for the conditions overlap or cross each other, we needed to use the median of the signed ANOVA (1-p)'s as the measure of differential expression. (When there is no overlap or crossing of profiles for the conditions, except for the sign of the median ANOVA (1-p), it does not matter whether we use the signed or unsigned methodology).

## Conclusion

Our first conclusion is that the median ANOVA (1-p) approach and its median signed ANOVA (1-p) variant presented here provide conceptually and computationally simple but effective measures of differential expression. Even though these methods do not clearly outperform existing methods, they perform reasonably well (as evidenced by Figures 4, 5, 6, 7, 8 and 9), and the associated probe set plots provide invaluable tools for those who want to look beyond summary measures in assessing differential expression. Furthermore, since the median is a very robust statistic it might be less sensitive than other methods to the redefinition of GeneChip probe sets that have been suggested and even implemented [10] by several authors.

The second conclusion is that if we wish to have effective test beds for assessing (or training) differential expression algorithms we cannot be satisfied with truths that have been established at the input side of the controlled experiments. If the spike-in data sets are to be critical tools in differential expression research then we must establish truth at the point in the processing chain at which the algorithms begin. To penalize a differential expression methodology for not finding a gene which is differentially spiked-in but not differentially expressed in the arrays, or vice versa, diminished the value of the spike-in experiments. The additions or deletions of spiked-in genes proposed by a few authors is a step in the right direction, but the real need it to have community-wide agreement on which genes are differentially expressed for which conditions, based on the contents of the CEL files rather than on the designs of the experiments. Biological resources will no doubt have a large role in predicting and explaining differences between the concentration-based "input truths" and the image-based "output truths" that differential expression tools work with. Nonetheless, the ultimate resolution of what is expressed and what is not expressed has to come from the CEL files themselves (if not the DAT that produced them).

**Figure 18:**
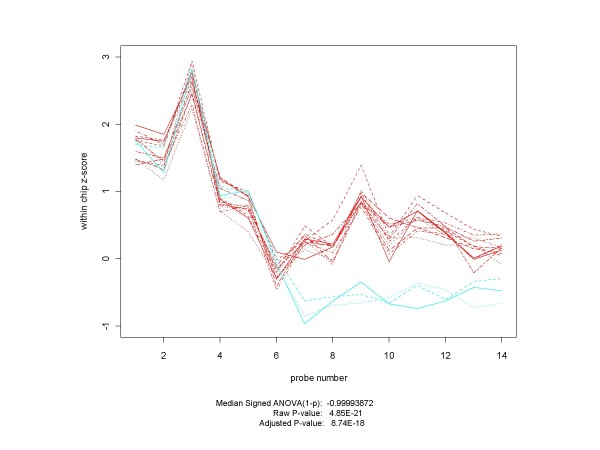
**Plot of gene with a possibly ambiguous differential expression status**. In this paper we point out the need to establish truth at the probe set/CEL file level as well as at the spike-in level. Achieving that goal will require community-wide consensus as to what constitutes differential expression, based on the contents of the probe sets involved in the comparison. For some genes consensus might not be an easy thing to achieve, as this plot of 1552_i_at from the HG-U95A experiment suggests. HG-U95A experimental condition Q is plotted in red, and condition A in cyan. Even though all summary measures considered in this paper declare this gene to be highly differentially expressed, it would not be at all surprising to find many opinions as to whether this gene is really expressed or not. Just as "Marginal" is an acceptable condition when making MAS 5 calls, so "Ambiguous" may have to be an acceptable state for some comparisons.

Determining truth at the CEL file level for all pairs of conditions will take a lot of work, but that is a requirement for a test bed that can be trusted to assess the effectiveness of the various differential expression paradigms. However, in practice there will probably be "only" several hundred comparative gene conditions that will require close examination of probe set profiles, and initially it makes sense to focus on the d = 1 conditions. It may well be that for some genes there will not be community-wide agreement as to whether they are differentially expressed or not for some pairs of conditions. Figure 18 presents a possible example of such a gene from the Q versus A conditions of the HGU-95A Latin Square design. Although the adjusted p-values for median signed ANOVA (1-p), RMA and PLM (8.7 × 10^-18^, 6.9 × 10^-12^, and 4.6 × 10^-16^, respectively) all strongly indicate differential expression, a biologist looking at the profile plots might well have second thoughts (either about differential expression or about the validity of the probe set itself). If researchers cannot agree if a collection of probe sets is differentially expressed or not, we cannot expect mechanized procedures to be in agreement either. In such cases, there may need to be a label in the CEL file truth metadata indicating the ambiguous status of the condition.

## Authors' contributions

There is a single author for this paper.
